# Transmembrane adaptor protein PAG1 is a novel tumor suppressor in neuroblastoma

**DOI:** 10.18632/oncotarget.8116

**Published:** 2016-03-16

**Authors:** Saurabh Agarwal, Rajib Ghosh, Zaowen Chen, Anna Lakoma, Preethi H. Gunaratne, Eugene S. Kim, Jason M. Shohet

**Affiliations:** ^1^ Department of Pediatrics, Section of Hematology-Oncology, Texas Children's Cancer Center, and Center for Cell and Gene Therapy, Baylor College of Medicine, Houston, Texas 77030, USA; ^2^ Department of Biology & Biochemistry, University of Houston, Houston, Texas 77204, USA; ^3^ Michael E. DeBakey, Department of Surgery, Division of Pediatric Surgery, Baylor College of Medicine, Houston, Texas 77030, USA; ^4^ Department of Surgery, Division of Pediatric Surgery, Keck School of Medicine, University of Southern California, Los Angeles, California 90027, USA

**Keywords:** neuroblastoma, Src, PAG1, tumor suppressor

## Abstract

(NB) is the most common extracranial pediatric solid tumor with high mortality rates. The tyrosine kinase c-Src has been known to play an important role in differentiation of NB cells, but the mechanism of c-Src regulation has not been defined. Here, we characterize PAG1 (Cbp, Csk binding protein), a central inhibitor of c-Src and other Src family kinases, as a novel tumor suppressor in NB. Clinical cohort analysis demonstrate that low expression of *PAG1* is a significant prognostic factor for high stage disease, increased relapse, and worse overall survival for children with NB. *PAG1* knockdown in NB cells promotes proliferation and anchorage-independent colony formation with increased activation of AKT and ERK downstream of c-Src, while *PAG1* overexpression significantly rescues these effects. In vivo, *PAG1* overexpression significantly inhibits NB tumorigenicity in an orthotopic xenograft model. Our results establish PAG1 as a potent tumor suppressor in NB by inhibiting c-Src and downstream effector pathways. Thus, reactivation of *PAG1* and inhibition of c-Src kinase activity represents an important novel therapeutic approach for high-risk NB.

## INTRODUCTION

Neuroblastoma (NB) is a highly aggressive pediatric malignancy and accounts for 13% of cancer morbidity in children with current cure rates less than 50% [1]. The MYCN oncogene is a primary driver of this malignancy and is also amplified in almost 50% of high-risk cases. Additional oncogenes including c-Src (cellular-Src) are known to increase NB invasiveness and proliferation in vitro [2, 3]. Here, we present in vitro, in vivo, and clinical data defining the major repressor of c-Src, PAG1 (Phosphoprotein Associated with Glycosphingolipid-enriched microdomains 1), as a novel tumor suppressor in high-risk NB.

PAG1 (Csk binding protein, Cbp) is a ubiquitous lipid-raft associated scaffold protein that inhibits Src Family Kinases (SFKs) including c-Src [4]. The activated transmembrane PAG1 binds and co-localizes both SFKs and Csk (C-terminal Src Kinase) to facilitate inhibition of SFKs by Csk. Thus PAG1 is essential for optimal Csk based repression of c-Src [5]. PAG1 can also inhibit c-Src independent of Csk by directly sequestering this kinase to lipid-rafts. PAG1 represses c-Src and prevents activation of multiple downstream effector proteins controlling cellular adhesion, proliferation, inflammation and differentiation [2, 6]. Importantly, negative feedback activation of PAG1 controls potent proliferative and oncogenic functions of c-Src in non-transformed cells [7, 8].

Elevated c-Src activity is found in multiple malignancies including NB that promotes proliferation, cell-cell adhesion, and anti-apoptotic functions [5, 9]. Small molecule tyrosine kinase inhibitors such as dasatanib and PP2 induce apoptosis and block proliferation of NB cells in culture [10, 11]. However, lack of specificity has made the dissection of the essential targets of these drugs difficult [12]. In addition, the endogenous mechanisms promoting aberrant c-Src activation in tumors remain poorly understood. Thus, our analysis of patient cohorts revealing a highly significant correlation of low PAG1 levels with poor survival, prompted us to further investigate the role of PAG1 in NB pathogenesis.

Herein, we demonstrate that PAG1 gain-of-function inhibits NB proliferation and colony formation in vitro, while xenografts with high PAG1 expression show limited tumorigenicity in vivo. These PAG1 effects correlate with inhibition of active c-Src (pY416) and downstream effector proteins AKT and ERK. Overall, our findings demonstrate potent tumor suppressor functions for PAG1 and suggest transcriptional repression of PAG1 is a major mechanism for c-Src hyperactivity in NB. These findings further suggest that reactivation of PAG1 may be a clinically significant novel therapeutic approach for NB.

## RESULTS

### PAG1 expression strongly correlates with overall survival of neuroblastoma patients

**Figure 1:**
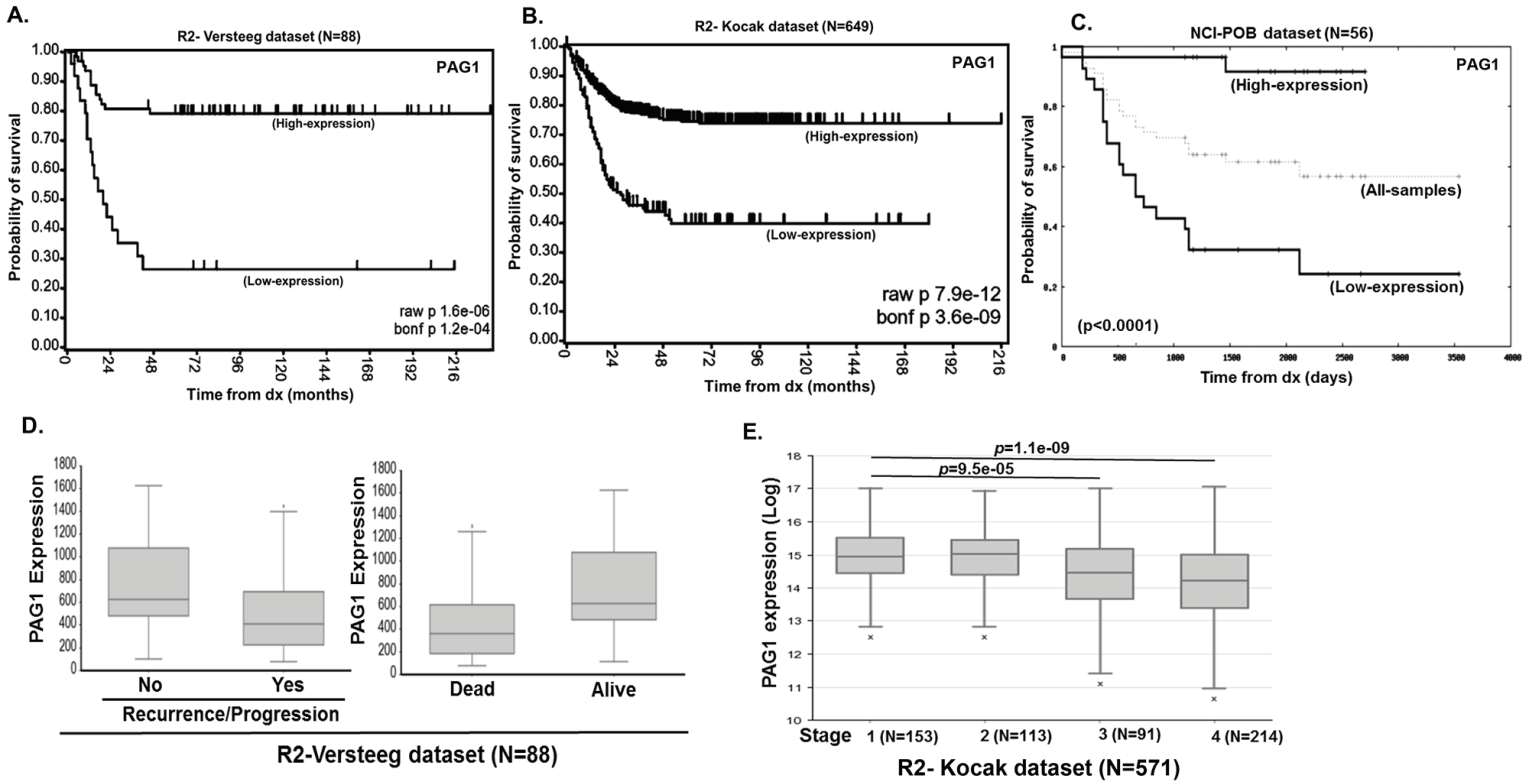
PAG1 is a prognostic factor in neuroblastoma. Kaplan-Meier curves shows the probability of overall survival based on PAG1 expression level **A.** 88 patients in the Versteeg dataset, **B.** 649 patients in the Kocak dataset and **C.** 56 patients in the NCI-POB dataset. **D.** R2-Versteeg dataset analysis showing correlation of PAG1 expression levels to neuroblastoma recurrence or progression and overall outcome. **E.** R2-Kocak dataset analysis showing correlation of PAG1 expression levels to neuroblastoma disease stages.

To evaluate how transcription of PAG1 correlates with NB outcomes, we analyzed multiple independent clinical cohorts of NB patients using the R2-data analysis platform. Kaplan-Meier analyses of datasets revealed that low PAG1 transcript levels strongly correlated with poor overall and event-free survival for the Versteeg cohort (n=88) (p<0.0001), for the Kocak dataset (n=476) (p<0.0001), and for the NCI-POB dataset (n=56) (p<0.0001) (Figure 1A, 1B, 1C). Other disease factors, such as relapse or tumor progression, and risk of death due to disease was also found to inversely correlate with PAG1 expression (p<0.001) (Figure 1D). In addition, higher stage, more aggressive tumors have significantly lower PAG1 expression suggesting that loss of PAG1 function may lead to de-differentiated invasive malignancy (Figure 1E). We observed a strong inverse correlation between PAG1 and MYCN expression levels (p<0.001) in all patient datasets analyzed (Supplementary Figure S1). MYCN non-amplified samples in clinical cohorts show worse overall survival with low PAG1 levels (p<0.001) while the MYCN amplified samples did not show significant correlation of PAG1 levels to overall survival (Supplementary Figure S2). This is due to aggressive disease progression in MYCN amplified tumors due in part to suppression of PAG1 (Supplementary Figure S1). These findings suggest that active suppression of PAG1 is an important factor influencing the biology and response to therapy of both MYCN amplified and non-amplified high-risk NB.

### PAG1 inhibits oncogenic potential of neuroblastoma cell lines

**Figure 2:**
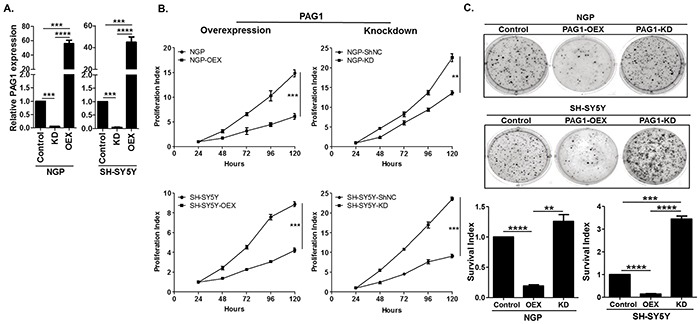
PAG1 is a tumor suppressor in neuroblastoma in vitro. **A.** Quantitative PCR (qPCR) analysis of PAG1 mRNA expression in PAG1 overexpression and knockdown cell lines in NGP and SH-SY5Y. Experiment was performed in triplicates and repeated thrice. Data represented as mean ± SD (T-test, ***p<0.001, ****p<0.0001). PAG1 mRNA expression in control cell lines NGP and SH-SY5Y was used to quantitate relative expression. **B.** Cellular proliferation assay of PAG1 gain and loss of function in NGP and SH-SY5Y NB cell lines. Cell proliferation was measured using MTS assay over 120 h. Proliferation index over the time was calculated by normalizing to the 24 h time point. Experiments were repeated three times with six replicates for each condition and represented as mean ± SD. (T-test, **p<0.01, ***p<0.001). **C.** Representative pictures of colony formation assay for PAG1 gain and loss of function in NB cell lines. Survival index shows the quantitation of relative inhibition of colony formation in different cell lines and is normalized to control or original cell line. Data represented as mean ± SD (T-test, **p<0.01, ***p<0.001, ****p<0.0001). OEX= PAG1-overexpression, KD= PAG1-knockdown, shNC= negative/mock control knockdown.

To further investigate the effect of PAG1 in NB, we derived gain-of-function and loss-of-function NB cell lines from the MYCN amplified cell line NGP and non-amplified cell line SH-SY5Y. Validation of these cell lines for PAG1 mRNA expression by qPCR assay shows profound increase of PAG1 levels in PAG1 over-expression (PAG1-OEX) cell lines and significant decrease of PAG1 levels in PAG1 knockdown (PAG1-KD) cell lines (Figure 2A). In MTS proliferation assays, PAG1-OEX decreases proliferation in both NGP and SH-SY5Y lines compared to parental cell lines (p<0.001). Furthermore, PAG1-KD increases cell proliferation in NGP by 1.6 fold (p<0.01) and in SH-SY5Y by 2.6 fold (p<0.001) (Figure 2B). In soft-agar colony formation assays, PAG1-OEX markedly decreases anchorage-independent colony formation (p<0.0001) while PAG1-KD increases the colony formation in NGP by 1.2 fold (p=ns) and in SH-SY5Y by 3.4 fold (p<0.001) (Figure 2C).

### PAG1 reduces neuroblastoma tumorigenicity in vivo

**Figure 3:**
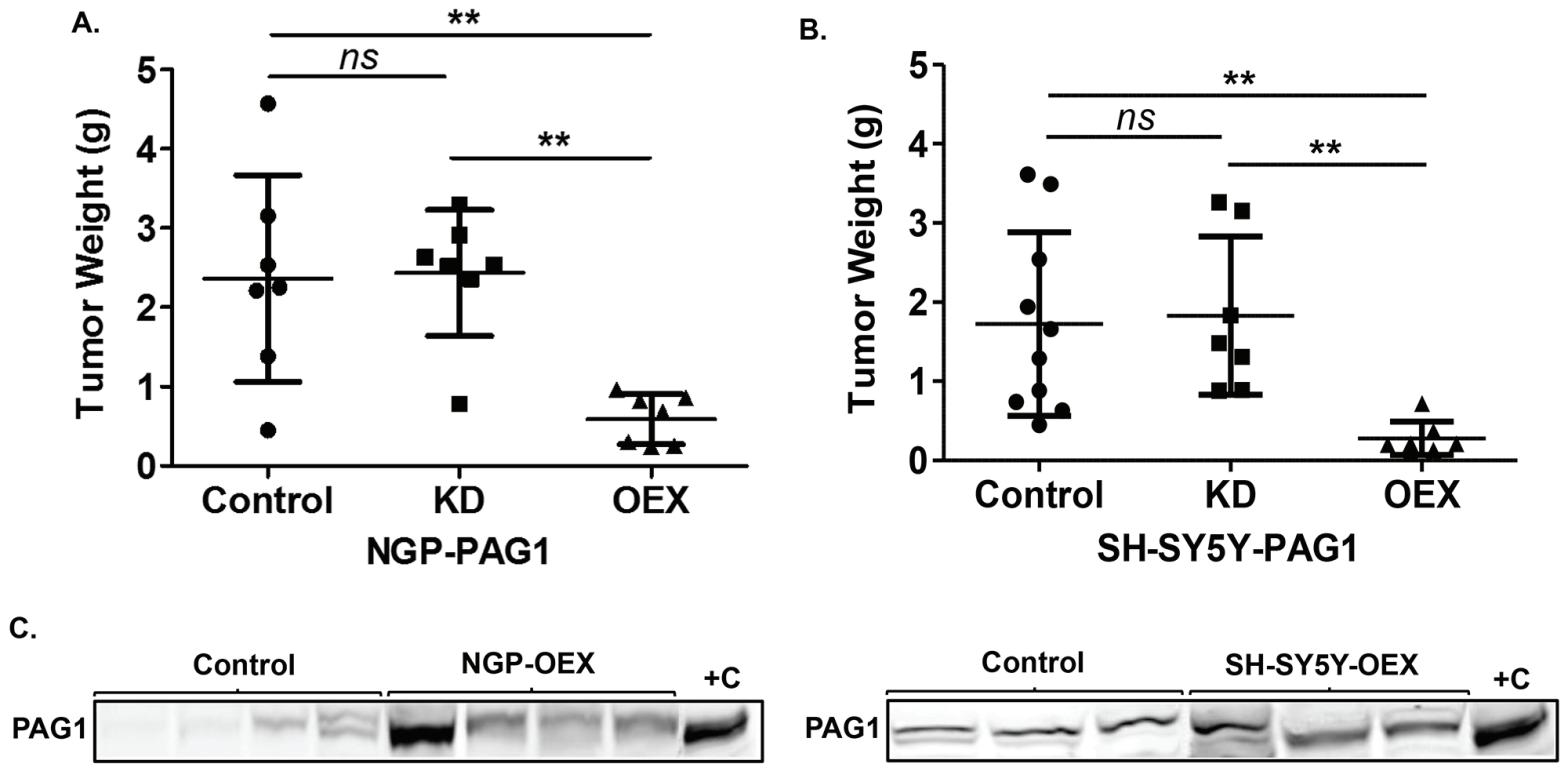
PAG1 is a tumor suppressor in neuroblastoma in vivo. **A.** Tumor xenografts of different PAG1 gain and loss of function cell lines in NGP and **B.** SH-SY5Y were developed and tumor weights were analyzed. Data represented for individual tumors in cohorts and as mean ± SD (Mann-Whitney test, ns= non significant, **p<0.01). **C.** Western immunobloting analysis of tumor samples shows overexpression of PAG1 in NGP-OEX and SH-SY5Y-OEX tumor xenografts in comparison to control NGP and SH-SY5Y xenografts respectively. Protein was isolated from flash frozen tumor tissues. +C represents positive control and is the protein isolated from respective OEX cell line. OEX= PAG1-overexpression, KD= PAG1-knockdown.

To evaluate the effects of PAG1 loss and gain-of-function on NB tumorigenicity in vivo, we generated cohorts of xenografts in athymic immunodeficient nude mice via orthotopic injection into the renal capsule as previously described [18]. Results show that both NGP and SH-SY5Y PAG1-OEX cell line cohorts have significant (p<0.01) reduction in tumor weights in contrast to parental control cell line and PAG1-KD cell line xenograft cohorts (Figure 3A, 3B). We further verified the expression levels of PAG1 in harvested tumor tissues, and as expected, observed increased PAG1 expression in PAG1-OEX xenograft tumor tissues compared to parental control cell line tumors (Figure 3C). These in vivo studies further validate our in vitro observations that PAG1 is able to serve as a potent tumor suppressor in NB.

### PAG1 inhibits c-SRC and downstream signaling pathways

**Figure 4:**
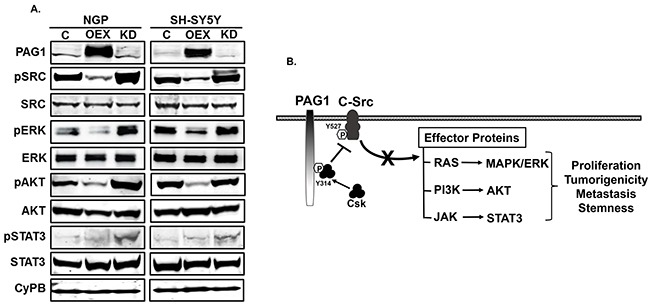
PAG1 inhibits c-Src and downstream effector proteins. **A.** Western blots of represented proteins in PAG1 overexpression (OEX), knockdown (KD) and control (C) NGP and SH-SY5Y cell lines. CyPB was used as a loading control for all blots, as it is not known and observed to be affected by PAG1 levels in NB cell lines. **B.** Mechanism of PAG1 action in neuroblastoma. PAG1 acts as an adaptor protein to recruit the Csk from cytoplasm at specific site Tyr-314 and bring it to close proximity with membrane bound c-SRC and other SFKs. Csk then directly inhibit c-Src and other SFks by phosphorylating the Tyr527 [5, 6]. c-Src is known to activate different cellular functions by controlling the effector proteins of different pathways (RAS, PI3K and JAK) either directly or indirectly [4]. Inhibition of c-Src by PAG1 leads to downstream inhibition of different cellular pathways that further translate in to reduction in overall proliferation and tumorigenicity, as confirmed by our in vitro and in vivo results in neuroblastoma.

In order to define the mechanism(s) of action for PAG1 as a tumor suppressor, we performed Western blot analysis for c-SRC and associated downstream signaling pathways. We observed that PAG1 over-expression in both NGP and SH-SY5Y cell lines significantly inhibits the phosphorylation and activation of the proto-oncogene c-SRC (phosphorylation at Tyr416). PAG1 over-expression also reduces phosphorylation of downstream RAS pathway effector proteins pERK, pAKT, and pSTAT3 (Figure 4A). In contrast, knockdown of PAG1 expression in both NGP and SH-SY5Y cell lines rescues these effects and induces phosphorylation and activation of c-SRC and downstream effector proteins (Figure 4A). An illustrated summary of the PAG1 tumor suppressor mechanism of action in NB is shown in Figure 4B. The transmembrane adaptor protein PAG1 recruits Csk and brings it in close proximity to active c-SRC. Csk then phosphorylates c-SRC at Tyr527 that leads to inhibition of c-Src kinase functions and consequently downstream effector protein signaling pathways [5].

### Transcriptional repression of PAG1 in neuroblastoma

**Figure 5:**
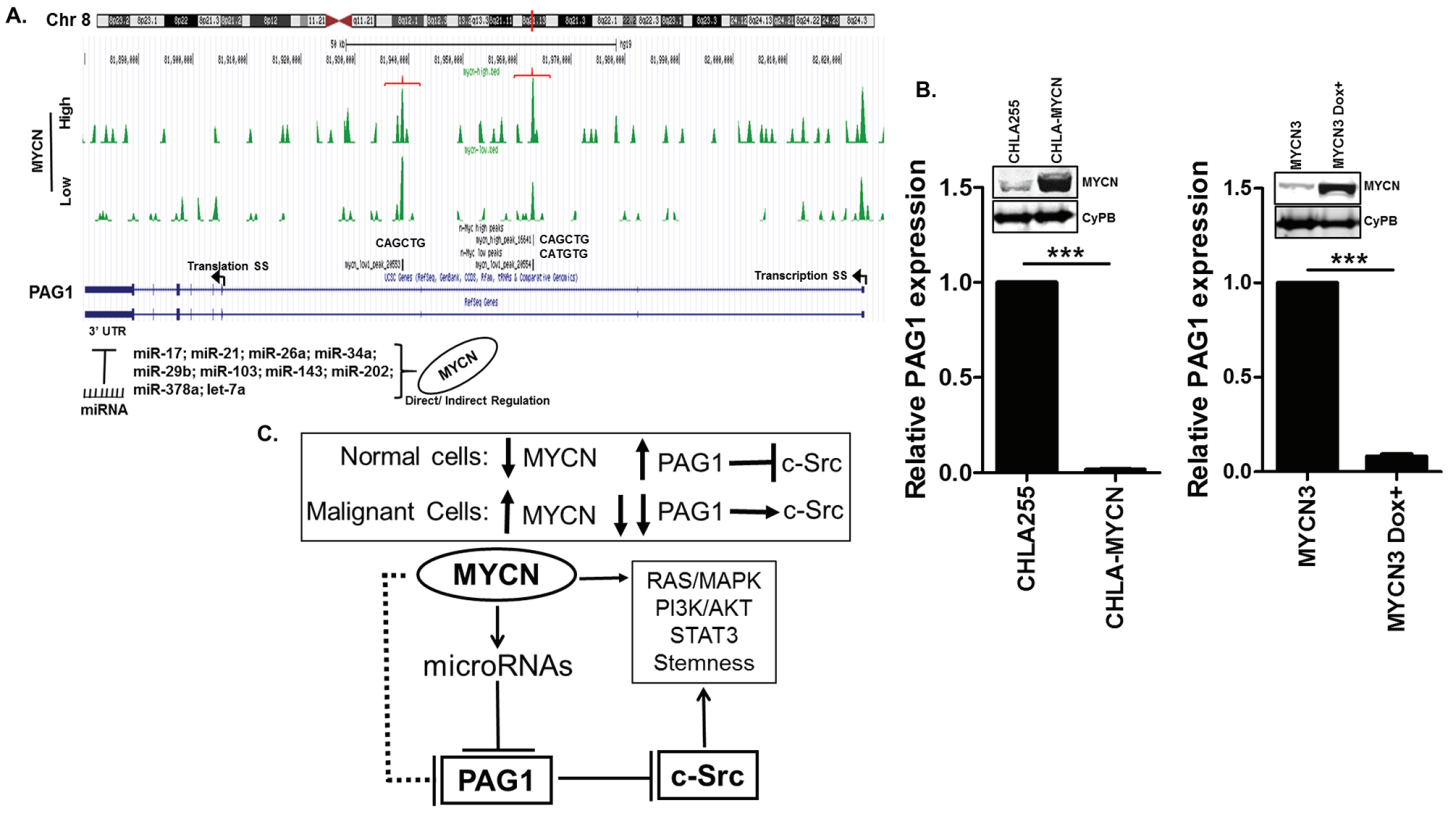
Transcriptional regulation of PAG1 in neuroblastoma. **A.** MYCN ChIP-seq database [16] analysis in MYCN high and low conditions at the PAG1 gene locus showing two binding sites with non-canonical e-boxes (brackets). Arrows represent the transcription and translation start sites on PAG1 gene. Analysis of PAG1 3′UTR shows binding sites of several miRNAs directly or indirectly activated by MYCN. **B.** Transcription profiling of PAG1 mRNA expression in two independent MYCN overexpression models in neuroblastoma. CHLA-MYCN cell line stably overexpress MYCN [17] and MYCN3 cell line express MYCN in response to doxycycline induction [16]. MYCN3 cell was either not treated or treated with doxycycline for 24 h. Western Immunoblot for MYCN was performed to validate the expression models and CyPB was used as loading control. PAG1 mRNA expression was analyzed by qPCR and represented as mean ± SD (T-test, ***p<0.001). Experiment was performed in triplicates and repeated thrice. PAG1 mRNA expression in control cell lines CHLA-255 and MYCN3 was used to quantitate relative expression. **C.** PAG1 repression contributes to neuroblastoma tumorigenesis. In normal non-malignant cells with normal PAG1 levels, c-Src activity is limited by PAG1/Csk induced negative phosphorylation. In malignant cells high levels of MYCN represses PAG1 that results in further c-Src activation. MYCN either directly or indirectly via miRNAs represses PAG1 expression. Inhibition of PAG1 leads to increased c-Src levels that further enhance activation of multiple oncogenic pathways including STAT3, PI3K and others.

We sought to determine whether PAG1 is directly inhibited by MYCN since MYCN is a primary oncogenic driver of NB and more aggressive tumors typically have increased MYCN expression [19]. MYCN-ChIP-seq data analysis for the PAG1 promoter and 5′UTR demonstrates lack of a canonical CACGTG E-box [16]. However, sequence analysis of the PAG1 5′UTR reveals two non-canonical binding sites (CAGCTG and CATGTG) suggesting that PAG1 could be directly repressed by the MYCN oncogene (Figure 5A). We also found binding sites of several microRNAs, directly or indirectly activated by MYCN in the 3′UTR of *PAG1* (Figure 5A and Supplementary Figure S3, S4), including sites for miR17a (a component of the miR-17-92a cluster) and multiple other microRNAs known to contribute to NB pathogenesis [16, 20, 21]. To validate that PAG1 is repressed by MYCN, we analyzed the PAG1 mRNA levels in two independent MYCN overexpression systems. Results shows significant reduction (<0.001) of PAG1 levels in stable MYCN overexpressing CHLA-255-MYCN cell line and inducible MYCN expressing MYCN3 cell line compared to control cell lines (Figure 5B). These findings and the lack of a canonical E-Box binding site suggest that MYCN indirectly represses PAG1 gene expression via its downstream microRNA targets or other mechanisms. We propose that MYCN represses PAG1 to promote c-Src activity and contributes to aggressive disease progression (Figure 5C).

## DISCUSSION

In summary, we demonstrate that PAG1 significantly alters NB tumor proliferation and xenograft formation. In addition, evaluation of NB patient cohorts demonstrates a significant inverse correlation of PAG1 expression with survival. Overexpression of PAG1 has also been shown to inhibit the growth of breast [22], lung [23], prostate [7] and colon cancer [24] primarily via repression of Src suggesting that repression of PAG1 may also be an important event in NB pathogenesis.

High stage NB displays elevated c-Src activity as well as increased downstream mTOR, PI3K/Akt and RAS/MAPK pathway activation [1, 19]. Earlier studies using tyrosine kinase inhibitors to block Src demonstrated sensitivity of NB cells to the non-specific Src inhibitors such as dasatanib [10], PP2 [11, 25] and other SFK inhibitors [3, 9, 26]. While these studies demonstrated in vitro proliferation arrest and apoptosis, the mechanisms for increased c-Src signaling in NB remained poorly defined. Here we demonstrate that increased expression of PAG1 decreases c-Src activation and inhibits tumor growth in vivo, and this correlates with reduced levels of pAKT and pERK.

PAG1 typically recruits Csk to co-localize and inhibit c-Src in lipid-rafts. Studies with Csk deficient cells demonstrated that PAG1 also inhibits Src through a secondary pathway by directly sequestering Src in lipid-rafts to prevent Src controlled transformation [24]. Additional PAG1 functions include Csk independent repression of RAS [27], binding with EBP5 [28], and regulating SOCS1–mediated ubiquitination and degradation of c-Src and other SFKs [29]. Thus accumulating evidence suggests that transcriptional regulation of PAG1 plays an important role regulating SFKs in cancer via multiple interactions. PAG1 is also reported to be controlled at epigenetic level in cancer cells, and treatment with a histone deacetylase (HDAC) inhibitor as well as by siRNA against HDAC1/2 restore PAG1 expression and activity [30]. We propose that antagomiRs or other approaches to block MYCN driven microRNAs that repress PAG1 transcription may present effective therapeutic strategies for high-risk NB.

There is increasing interest in developing specific inhibitors to c-Src and other non-receptor Src related tyrosine kinases due to the plethora of ascribed oncogenic functions [31]. NDRG1, a suppressor of metastasis is recently shown to inhibit c-Src and downstream signaling pathways in three tumor cell-types [32]. Metastasis represents a major cause of morbidity and mortality for NB and identification of upstream regulator of c-Src, PAG1 provides rationale for novel therapeutic strategies to inhibit this oncogene. The elucidation of the pathway(s) that lead to tumor suppressor PAG1 down-regulation may shed light on the mechanisms of NB tumor progression, and pave the way for developing new therapeutic interventions for children suffering with this deadly malignancy.

## MATERIALS AND METHODS

### Clinical patient cohorts

NCI-POB dataset include 56 primary patient tumors and their microarray gene expression profiling and clinical outcomes. This dataset is available from Oncogenomics platform (http://home.ccr.cancer.gov/oncology/oncogenomics). Versteeg dataset includes 88 and the Kocak dataset includes 649 [13] unique primary tumor profiles and are publically available from the gene expression databases at the R2: Genomic Analysis and Visualization Platform (http://hgserver1.amc.nl/cgi-bin/r2/main.cgi) [14].

### Cell culture

Human neuroblastoma cell lines (NGP, SH-SY5Y, CHLA-255, MYCN3, CHLA-255-MYCN) were routinely maintained and cultured as described previously [15, 16]. CHLA-255-MYCN cell line was courtesy provide by Dr. Leonid Metelitsa, Baylor College of Medicine [17]. All cell lines were validated for CD56 expression and by genotyping within the past 12 months. All cell lines used in this article were routinely tested for Mycoplasma on a monthly basis.

### Generating stable PAG1 overexpression and knockdown cell lines

Lentiviral vector plasmids EX-T0343-Lv105-B (Genecopeia, MD) and pGIPZ-PAG1-shRNA set (Thermo Fisher Scientific, MA) were used to transduce NB cell lines for PAG1 overexpression and knockdown as described previously [15]. Set of pGIPZ-PAG1-shRNA include four shRNA vectors and testing with transient transfection in NB cell lines shows that shRNA2 (mature antisense sequence: TCTCTTGTTAGTTTCACTG) efficiently knockdown PAG1, therefore we used this shRNA vector for further study. Non-silencing pGIPZ-control-shRNA vector was used to generate NGP-shNC and SH-SY5Y-shNC cell lines as a control.

### Cell proliferation and soft agar colony formation assay

Cell proliferation assay was performed using CellTiter 96® Cell Proliferation Assay (G4000, Promega) according to manufacturer's instructions and as described previously [15]. Proliferation index was calculated by normalizing each reading to the 24 h time point. Colony formation soft agar assays were performed using standard conditions as described previously [15]. All assays were performed in triplicates and repeated twice with proper controls.

### PAG1 quantitative PCR assay

PAG1 mRNA expression in different cell lines were measured using quantitative real-time RT-PCR assay. Extraction of RNA, cDNA synthesis and qPCR was performed as described previously [18]. Assays were performed in triplicates for each sample using Power SYBR Green PCR Master Mix (Applied Biosystems) and normalized to GAPDH. Primers used were, PAG1-F 5′ATCACCCTGTGGGGAAGTCT3′ and PAG1-R 5′TCCTTGTCTGAAGGCACGTT3′, GAPDH-F 5′GGTCGTATTGGGCGCCTGGTC3′ and GAPDH-R 5′GCCAGCATCGCCCCACTTGA3′. Dissociation curves were analyzed for primer pairs as a means to ensure the quality of amplicon and to monitor primer dimers.

### Mice and xenograft assay

Four to six week-old female inbred athymic immunodeficient Nude mice (Nu/Nu) (Taconic Biosciences, NY) were used for all xenograft studies. Mice were implanted using a previously described orthotopic xenograft model of NB [15]. Briefly, 1×10^6^ NB cells were surgically implanted in the sub-renal capsule of mice, and tumor growth was monitored bi-weekly by bioluminescent imaging (IVIS Lumina XR System, Caliper Life Sciences, MA). After 28 days of implantation all mice were euthanized and tumors were resected and weighed. All animal experiments are approved by the nstitutional animal care and use committee.

### Western blot

Total and phospho- proteins were extracted by lysing cells in RIPA buffer supplemented with protease inhibitor cocktail (Complete mini EDTA free, Roche) and phosphatase inhibitor cocktail (PhosSTOP, Roche). Extracted proteins were analyzed by Western blotting as described previously [15]. Primary antibodies for PAG1 (MAB5285, R&D systems), MYCN (OP13L, EMD Millipore), pSRC (6943), SRC (2102), pERK (4370), ERK (4695), pAKT (4060), AKT (9272), pSTAT3 (9145), STAT3 (4904) were purchased from Cell Signaling Technology, MA, and loading control CyPB antibody (sc20361) from Santa Cruz Biotech, TX.

### Statistical analysis

Data values for *in vivo* experiments are expressed as mean ± SEM and compared using Mann-Whitney (two-tailed nonparametric analysis) test. Student's t-test (two-tailed or one-tailed distribution with unequal variance) was applied to compare the results shown for *in vitro* experiments unless otherwise stated. Assays were performed in triplicate and repeated. Correlation between PAG1 and MYCN expression in human NB patients was examined with Pearson's correlation. Survival analyses were performed using Kaplan-Meier method and two-sided log-rank tests. A p-value less than 0.05 was considered significant.

## SUPPLEMENTARY FIGURES


