# Supplementary material for: Transmembrane adaptor protein PAG1 is a novel tumor suppressor in neuroblastoma
Source: Oncotarget. 2016 Mar 16;7(17):24018–26. doi: 10.18632/oncotarget.8116 (PMC5029681; doi:10.18632/oncotarget.8116)
Supplement: Supplementary file 1 [file oncotarget-07-24018-s001.pdf]

SUPPLEMENTARY FIGURES

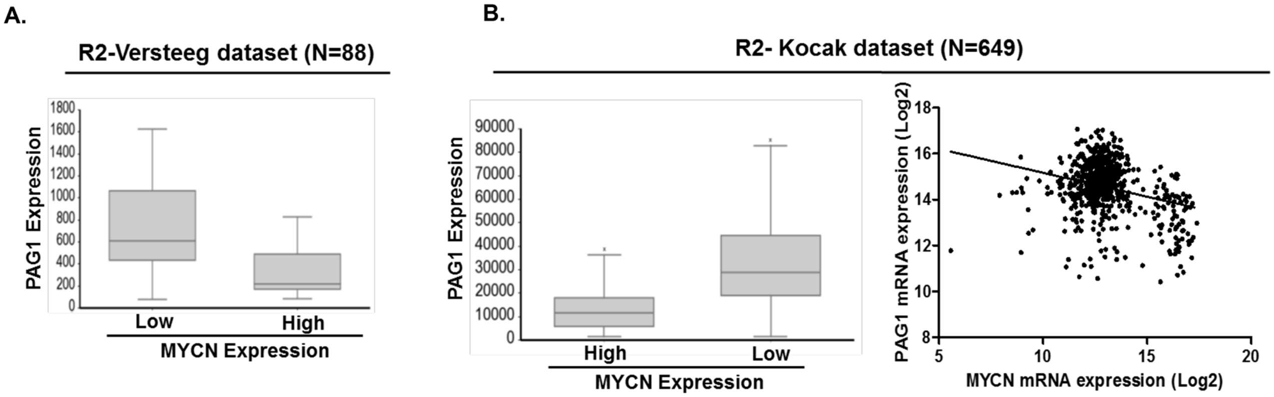

Supplementary Figure 1: A. R2-Versteeg dataset analysis and **B.** R2-Kocak dataset including 649 neuroblastoma patients showing correlation of MYCN and PAG1 mRNA expression. Two-sided Pearson's correlation was employed to analyze correlation.

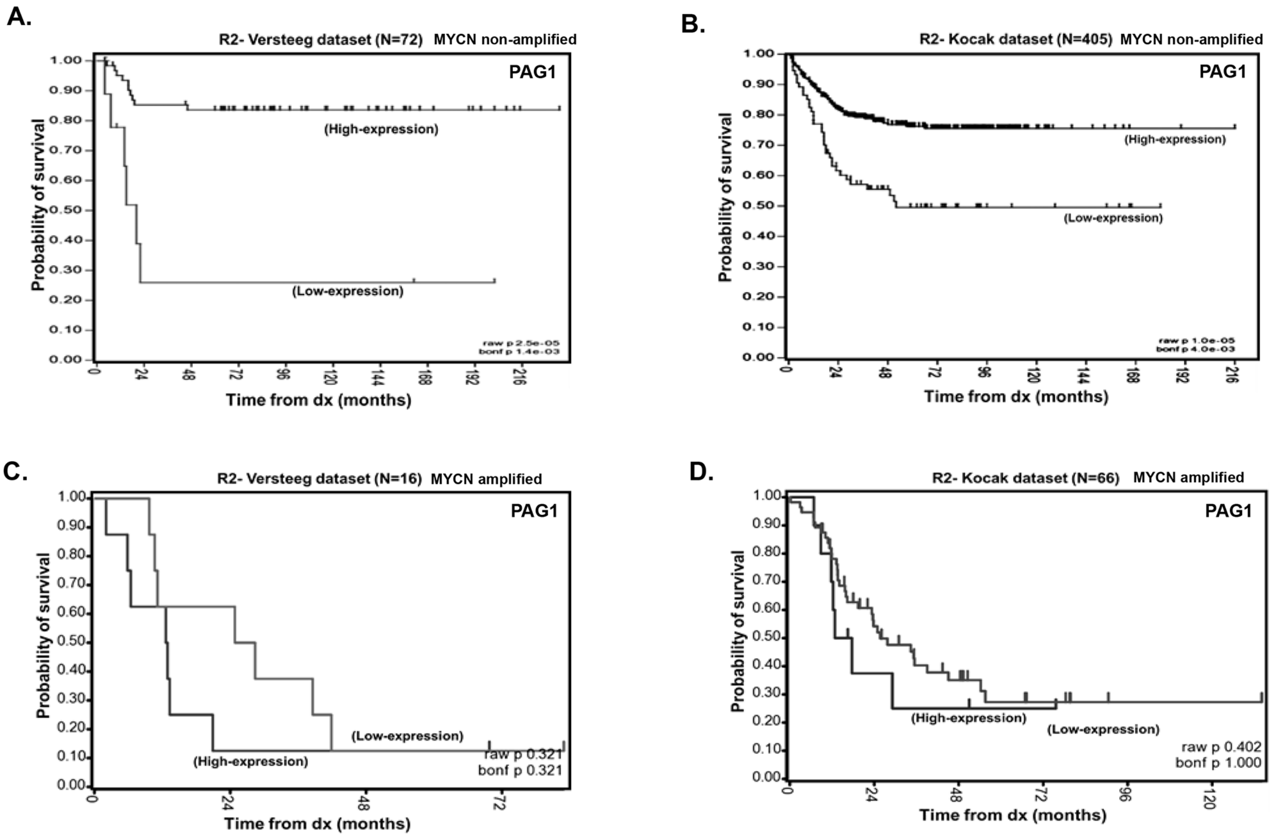

Supplementary Figure 2: A. Overall survival analysis of MYCN non-amplified neuroblastoma tumor samples from R2-Versteeg dataset (n=72) and **B.** R2-Kocak dataset (n=405). **C.** Overall survival analysis of MYCN amplified neuroblastoma tumor samples from R2-Versteeg dataset (n=16) and **D.** R2-Kocak dataset (n=66).

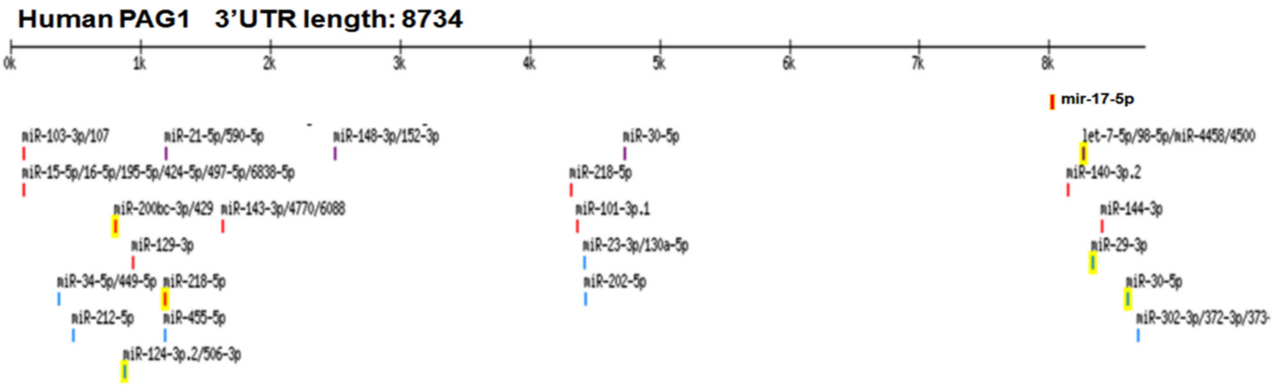

Supplementary Figure 3: TargetScan miRNA target prediction analysis showing target sites of different miRNAs on PAG1 3'UTR.

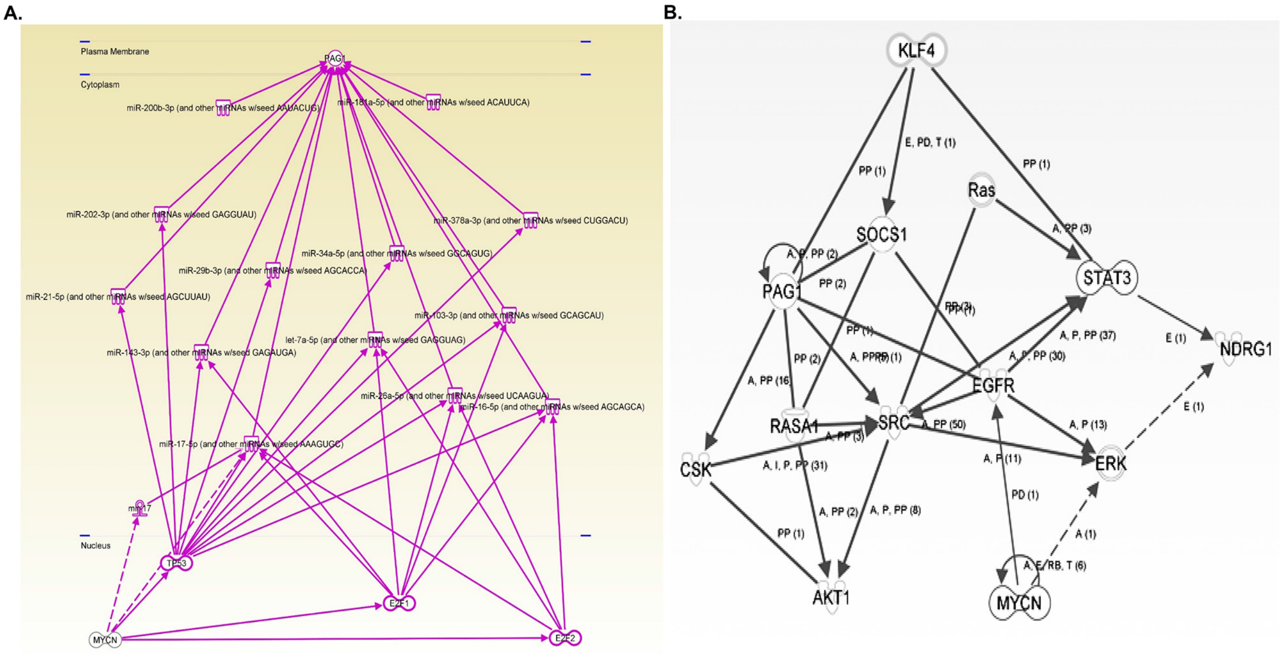

Supplementary Figure 4: A. Ingenuity pathway analysis for miRNAs target site prediction and regulation. Several miRNAs targeting PAG1 and their target sequence is shown. Most of the miRNAs are regulated by MYCN either directly (miRNA 17a-5p) or indirectly. **B.** Key regulators of neuroblastoma and PAG1 were analyzed for possible molecular interactions using Ingenuity pathway analysis.
